# Biosynthesis of aliphatic plastic monomers with amino residues in *Yarrowia lipolytica*

**DOI:** 10.3389/fbioe.2022.825576

**Published:** 2023-01-11

**Authors:** Gyuyeon Park, Ye Chan Kim, Minjeong Jang, Hyuna Park, Hong-Weon Lee, Wooyoung Jeon, Byung-Gee Kim, Kwon-Young Choi, Jungoh Ahn

**Affiliations:** ^1^ Department of Bioprocess Engineering, University of Science and Technology (UST) of Korea, Daejeon, South Korea; ^2^ School of Chemical and Biological Engineering, Seoul National University, Seoul, South Korea; ^3^ Biotechnology Process Engineering Center, Korea Research Institute of Biosceince and Biotechnology (KRIBB), Daejeon, Chungcheongbuk-do, South Korea; ^4^ Department of Environmental Engineering, College of Engineering, Ajou University, Suwon, Gyeonggi-do, South Korea; ^5^ Department of Environmental and Safety Engineering, College of Engineering, Ajou University, Suwon, Gyeonggi-do, South Korea

**Keywords:** α,ω-diamine, ω-amino fatty acids, ω-oxidation, ω-transamination, *Yarrowia lipolytica*, metabolic engineering

## Abstract

**Introduciton:** The α,ω-diamines (NH_2_-(CH_2_)_n_-NH_2_) and ω -amino fatty acids (NH_2_-(CH_2_)_n_-COOH) have been widely used as building blocks in polymerindustries. Medium- to long-chain (C_8_ to C_18_) fatty acid monomers with amino residues are almost exclusively produced *via* chemical processes that generate hazardous waste and induce severe environmental problems, such as global warming and pollution. Here, we present the construction platformstrains of *Yarrowia lipolytica* a cheese-ripening yeast, for direct biotransformation of hydrocarbons into medium- to long-chain α,ω-diamines and ωamino fatty acids using metabolic engineering of endogenous fatty acid ω- and β-oxidation pathways and introducing heterologous ω-transaminase in *Y. lipolytica*.

**Methods:** We deleted six genes encoding the acyl-CoA oxidase (ACO1–6) and four fatty aldehyde dehydrogenase genes (FALDH1-4), which catalyze fatty acid β-oxidation and downstream oxidation of fatty aldehydes in *Y. lipolytica*, respectively. The ω-transaminase from *Chromobacterium violaceum* DSM30191 was introduced into the genome of the Δ*POX* Δ*FALDH* strain under the control of *Y. lipolytica*-derived EXP1 promoters.

**Results and Discussion:** The Δ*POX* Δ*FALDH* strains with ω-CvTA successfully accumulated the corresponding C12 αω-diamines into a shaking culture medium with dodecane or dodecanol. In addition, these strains accumulated C_12_ ω-amino fatty acids from dodecanoic acid. With the commercially available α,ω-diacid bioprocess, this yeast biosynthesis producing medium- and longchain α,ω-diamines and ω-amino fatty acids could complete the yeast platform technology generating all medium- and long-chain aliphatic polyamide monomers, α,ω-biofunctionalized with one or both carboxylic acid and amino residues.

## 1 Introduction

Plastics are materials that possess a high molecular weight and are composed of repeating organic molecules (monomers). Plastics play essential and ubiquitous roles in modern life. Synthetic plastics are commonly produced *via* polymerization of petrol-based chemicals; this process consumes approximately 8% of the annual petroleum production (Carpenter 2020).

Polyester (PE) and polyamide (PA) plastics are the two most important classes of commercial plastics, and the monomers of each plastic are joined by an ester (-O-CO-) and an amide (-CO-NH-) linkage, respectively. Both plastics can be synthesized as homopolymers (e.g., PA12 laurolactam, polylactic acid from lactic acid) or copolymers (e.g., PA 12.12 from 1,12-diaminododecane, dodecanedioic acid or polybutylene succinate from succinate and 1,4-butanediol). For PA, the monomers are α,ω-fatty dicarboxylic acids (α,ω-diacids) and α,ω-fatty diamines (α,ω-diamine) or ω-amino fatty acids (ω-AFA), while for PE, the monomers are α,ω-diacids and α,ω-fatty dialcohols (α,ω-diol) or ω-hydroxy fatty acids (ω-HFAs) (Schaffer and Hass 2014). Compared to the short-chain (C_2_–C_6_) plastics, medium-chain (C_8_–C_14_) repeating monomers exhibit high-performance properties, including flexibility, chemical resistance, low moisture absorption, and resistance to stress cracking (Supplementary Figure S1A) (Albanese et al., 2016). At present, C_12_ monomers for aliphatic PA and PE are almost exclusively produced *via* chemical processes, which have several disadvantages, including limitations in the range of products, use of multi-step conversion processes (Supplementary Figure S1B), dependence on non-renewable feedstocks, and generation of hazardous waste and emissions, which induce severe environmental problems, such as global warming and pollution.

Biotechnology offers innovative approaches to overcome the disadvantages involved in the chemical processing of medium-chain polymeric monomers. Yeast biocatalysts can oxidize the terminal (ω) carbons of medium- to long-chain *n*-alkanes, fatty alcohols, and fatty acids *via* the ω-oxidation pathway (Craft et al., 2003; Van Bogaert et al., 2011), thereby converting the compounds to their corresponding α,ω-diacids or ω-hydroxy fatty acids (ω-HFAs). The yeasts *Candida tropicalis* and *Y. lipolytica* have been used as industrial biocatalysts to produce lipophilic products. *Y. lipolytica* is classified and genetically recognized as safe (GRAS) and can utilize the ω-oxidation pathway to oxidize hydrophobic substrates, generating corresponding α,ω-bifunctional metabolites (α,ω-diacids, α,ω-diol, and ω-hydroxy fatty acids) that can be used as polymer building blocks (Figure 2A). For plastic monomers with amino residues such as α,ω-diamines and ω-AFA, ω-oxidation reactions are connected to ω-transamination. Several studies have been performed to produce ω-AFAs from fatty acids using *Escherichia coli* expressing multi-enzyme cascade pathways, including heterologous monooxygenases and ω-transaminase (Ladkau et al., 2016; Ahsan et al., 2018; Ge et al., 2020). Sung et al. (2018) produced ω-AFAs from hydroxy fatty acids or α,ω-diamines from α,ω-diols using *Escherichia coli* containing heterologous alcohol dehydrogenase and ω-transaminase. Recently, Citoler et al. (2019) reported the biocatalytic amination of fatty acids through a one-pot tandem cascade catalyzed by a carboxylic acid reductase (CAR) and a transaminase (ω-TA). However, the use of yeast in synthesizing medium- to long-chain α,ω-diamines or ω-AFAs from low-cost raw materials has not yet reached its proof-of-concept stage.

Here, we present the construction of platform strains of *Y. lipolytica*, a cheese-ripening yeast, for direct biotransformation of hydrocarbons into medium- to long-chain α,ω-fatty diamines [NH_2_-(CH_2_)_n_-NH_2_] and ω-amino fatty acids [NH_2_-(CH_2_)_n_-COOH], which are building blocks for high-performance polymers (Carpenter 2020), *via* metabolic engineering of endogenous fatty acid ω- and β-oxidation pathways and introducing heterologous ω-transaminase (ω-TA).

## 2 Materials and methods

### 2.1 Yeast strains and plasmids

*Y. lipolytica* strains, plasmids, and oligonucleotides used in this study are listed in Supplementary Table S1. Genetic engineering was performed according to the standard procedures. All oligonucleotides were synthesized by Cosmogenetech Co., Ltd., (Daejeon, South Korea). Plasmids and DNA fragments were prepared using Qiagen kits (Germany), and other chemicals were purchased from Sigma-Aldrich (United States).

### 2.2 Genome engineering of *Y. lipolytica*

**FIGURE 1:**
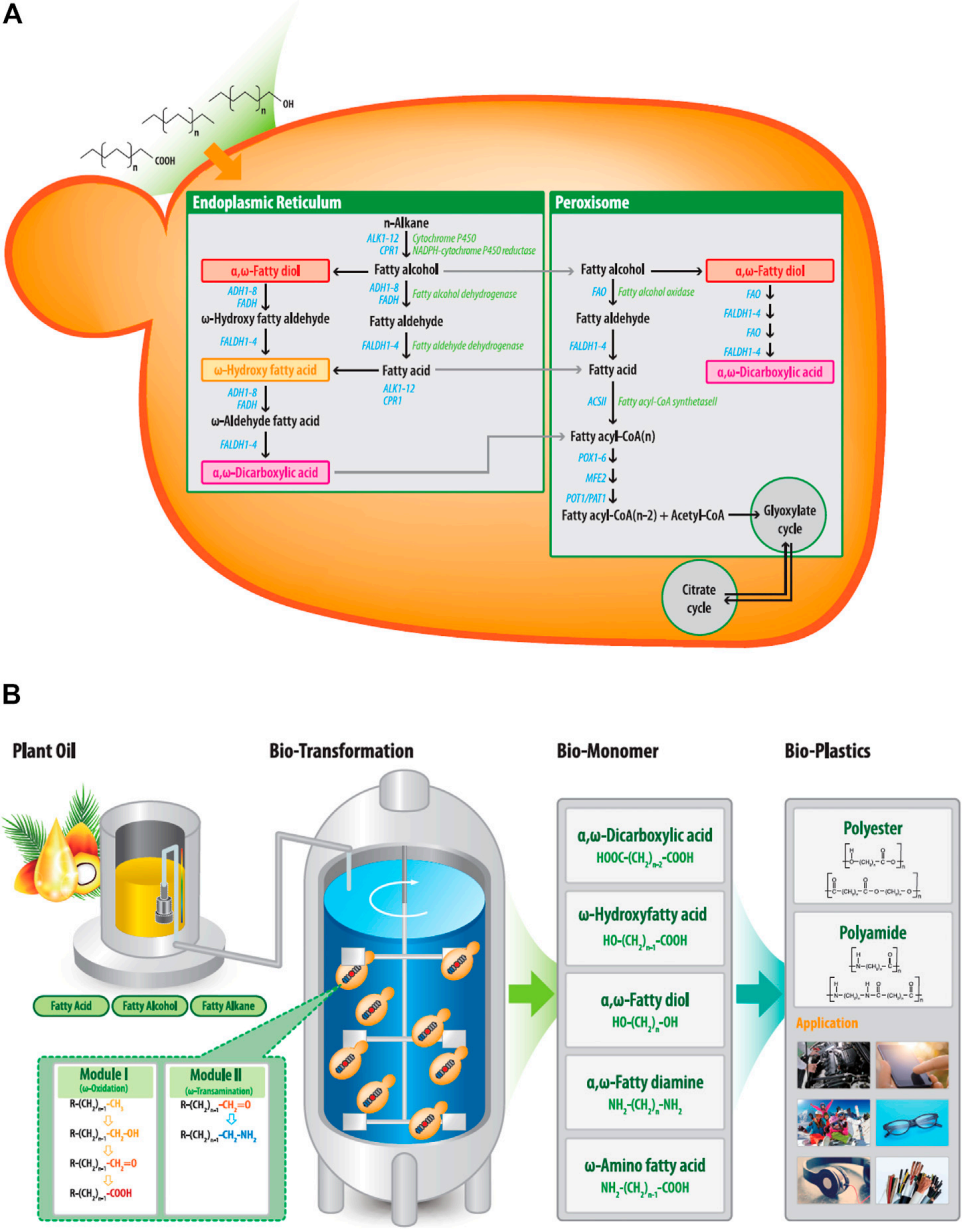
**(A)** Metabolic pathways for hydrophobic substrates, including fatty alkanes, fatty alcohol, and fatty acids, in *Y. lipolytica*. The substrates were removed in the cell and then converted to their corresponding fatty acids or α,ω-diacids *via* the ω-oxidation pathway. Fatty acids and α,ω-diacids are then degraded to acetyl-CoA during β-oxidation pathway. **(B)** Biotechnological synthesis of α,ω-bifunctional polymeric monomers from plant oil-based derivatives. Fatty alkanes, fatty alcohol, and fatty acids are used as feedstock and are converted to corresponding α,ω-bifunctional chemicals through module 1 and/or 2. These final products can be used as building blocks of polyester and polyamide (R: H_3_C-, HO-, O=, HOOC-).

Genome modification of *Y. lipolytica* was based on the homologous recombination and 5-fluoroorotic acid (5-FOA) selection using *URA3* as a recyclable selection marker (Figure 1). *URA3* was amplified from *Y. lipolytica,* and pop-out regions were used with glutamate production gene or histidine-related gene from the pGEM T easy cloning vector (Table 1) using the PCR using primers listed in Supplementary Table S2. To construct the gene disruption cassettes, the 5′- and 3′-flanking regions of each gene listed in Supplementary Table S2 were amplified from the genomic DNA of *Y. lipolytica* through PCR using the *XXX*_F1/*XXX*_R1 and *XXX*_F2/*XXX*_R2 primers (Supplementary Table S1). These homologous regions for the deletion and pop-out cassettes were obtained using overlap PCR, and the bipartite fragments were transformed into yeast cells by using a one-step transformation method. The 5-FOA selection and uracil marker rescue were performed. The successful disruption of a gene was verified using PCR using the primers *XXX*_F1/*XXX*_R1 and *XXX*_F2/*XXX*_R2. To introduce ω-transamination into *Y. lipolytica,* ω-transaminase (ω-TA) from *C. violaceum* DSM30191 was expressed under the control of *Y. lipolytica-*derived EXP1 promoters (Blazeck et al., 2011) with or without signal sequences from the *ALK1* at the N-terminus for ER membrane anchoring (Iida et al., 1998) (atg​tcc​aac​gcc​ctc​aac​ctg​tcg​ctg​gcg​ctc​ggc​gtc​ttt​ctg​cta​gcc​tac​tat​ggc​ttc​tcc​gtg​atc​cag​tac​cgc​atc​aaa​a ccc​gca​agc​tcg​aaa​aga​agt​gga​agt​gtg​gta​agc​cca​agg​ata​ttt​cac​gat​tc). Each ω-TA cassette was integrated into the FALDH4 locus of the engineered *Y. lipolytica* strain. Genomic DNA from the transformants was extracted using the Gene All DNA extraction kit (Gene all biotechnology, Korea). A Biometra T3000 thermocycler with Prime Star DNA polymerase (Takara, Japan) was used for PCR amplification. Amplified fragments were purified using the QIAgen purification kit (Qiagen, Germany), and DNA fragments were recovered from the agarose gels using the QIAquick gel extraction kit (Qiagen, Germany).

**TABLE 1:** *Y. lipolytica* strains and plasmids used in this study.

Strains	Genotype	
Y1-1	MatA, leu2-270, ura3-302::△leu2	
Y1-2	MatA, leu2-270, ura3-302::△leu2△ura3	
Y1-3	MatA, leu2-270, ura3-302::△leu2△ura3△ku70	
Y2-1	MatA, leu2-270, ura3-302::△ura3△ku70△pox1-6::LEU2, URA	
Y2-2	MatA, leu2-270, ura3-302::△ura3△ku70△pox1-6△faldh1-4::LEU2, URA	
Y2-3	MatA, leu2-270, ura3-302::△ura3△ku70△pox1-6△faldh1-4△fao::LEU2, URA	
Y3-1	MatA, leu2-270, ura3-302::△ura3△ku70△pox1-6△faldh1-4::Exp1p^a^ ω-CvTACytosol, URA	
Y3-2	MatA, leu2-270, ura3-302::△ura3△ku70△pox1-6△faldh1-4::Exp1p^a^ ω-CvTAER, URA	
Y4-1	MatA, leu2-270, ura3-302::△ura3△ku70△pox1-6△faldh1-4△fao△adh1-8△fadh△alk3::LEU2, URA	
Y4-2	MatA, leu2-270, ura3-302::△ura3△ku70△pox1-6△faldh1-4△fao△adh1-8△fadh△alk6::LEU2, URA	
Y4-3	MatA, leu2-270, ura3-302::△ura3△ku70△pox1-6△faldh1-4△fao△adh1-8△fadh△alk3△alk6::LEU2, URA	

Plamids	Backbone plasmids	Description
pYUHisG1	pGEM T easy	*URA3* maker, HISG1 pop-out cassette
pYUHisG2	pGEM T easy	*URA3* maker, HISG2 pop-out cassette
pYUGlt2	pGEM T easy	*URA3* maker, Glt2 pop-out cassette
pYUGlt3	pGEM T easy	*URA3* maker, Glt3 pop-out cassette
pYTEXP1	pTOP blunt	EXP1 promoter, ω-transaminase, *URA3* maker, Glt2 pop-out cassette

a EXP1p is the endogenous promoter from *Y. lipolytica*.

### 2.3 Biotransformation condition

A single colony of the engineered *Y. lipolytica* strains was pre-grown in a test tube containing 3 ml yeast extract Bacto-pepton dDextrose (YPD) medium overnight at 30°C with shaking at 200 rpm. One milliliter of pre-cultured broth was inoculated into a 500 ml baffled flask containing 100 ml of growth medium (pH 6.0) containing 50 g/L glucose, 10 g/L yeast extract, 6.7 g/L yeast nitrogen base without amino acids, 5 g/L (NH_4_)_2_SO_4_, and .05 g/L uracil. The cultures were incubated overnight at 30°C with rotation at 200 rpm. When an OD_600_ of 40–50 was reached, 50 ml of the culture broth was transferred to 250 ml baffled flasks containing 50 ml of the conversion medium (pH 7.6) containing 30 g/L glucose, 3 g/L yeast extract, 6.7 g/L YNB w/o amino acids, 5 g/L (NH_4_)_2_SO_4_, .05 g/L uracil, and 1 g/L substrate (fatty alkane, fatty alcohol, or fatty acid) with .05% Tween 80. This biotransformation was performed for 2 days at 30°C in a shaking incubator (200 rpm). To investigate the effect of multiple gene knock-out on cell growth, *Y. lipolytica* was incubated in a yeast extract-peptone medium (YPD) containing 10 g/L yeast extract and 20 g/L Bacto-peptone in sterile 24-well PS plates (Falcon, Corning, NY). Each well contained 400 µl of YP media with 2 µl of the pre-cultured *Y. lipolytica.* The *Y. lipolytica* culturing was done in 20 ml of YPD medium in 250 ml baffled flask incubated overnight at 30°C. The plate was incubated on a shaking plate reader (Tecan, Männedorf, Switzerland) at 30°C at 200 RPM, and the turbidity of each well was measured every 15 min (no sampling of the culture).

### 2.4 Analytical methods

The biomass was measured using a UV spectrophotometer (Uvikon XL, Secomam, France) at 600 nm absorbance. To analyze the subcellular localization of ω-*Cv*TA_ER_ and ω-*Cv*TA_cytosol_ introduced into *Y. lipolytica*, cells were prepared, and fluorescence microscopy observations were performed. PDI monoclonal antibody (MA1-10032) was used as the primary antibody for ω-*Cv*TA (Abfrontier company, Seoul, Republic of Korea) for the ER marker, and the goat anti-mouse IgG1 (H&L; Alexa Fluor^®^ 488) was used as the secondary antibody. Substrates and products were identified and quantified using gas chromatography-mass spectrometry (GC-MS) (Agilent GC/MSD 5975 coupled with an Agilent 7890A) equipped with a quadrupole mass selective detector on electroionization (EI) operated at 70 eV. An Agilent HP-5MS column (30 m length, .25 mm inner diameter, .25 μm film thickness) was used at a 10:1 split ratio, helium was used as a carrier gas, and the oven temperature was 150°C–172°C. The substrates and products were extracted and prepared for GC-MS analysis according to the corresponding methods shown in Supplementary Figure S2. Briefly, extraction was performed from acid-or base-treated culture broth using an equivalent volume of diethyl ether or chloroform. Methanol dissolved in 2.2% hydrochloric acid and 10% toluene was added to the reacted samples (1:1, v/v) and the mixture was incubated for 1 h at 100°C. After heating, the mixture was acidified with H_2_SO_4_ to convert the ionized ω-amino fatty acid to ω-amino fatty acid and directly extracted with diethyl ether. For sialylation, N,O-bis(trimethylsilyl)trifluoroacetamide was mixed with the extracted samples (1:1, v/v) and the mixture was incubated for 20 min at 60°C. Tetradecane was used as an internal standard.

## 3 Results

### 3.1 Genome engineering of *Y. lipolytica*

**FIGURE 2:**
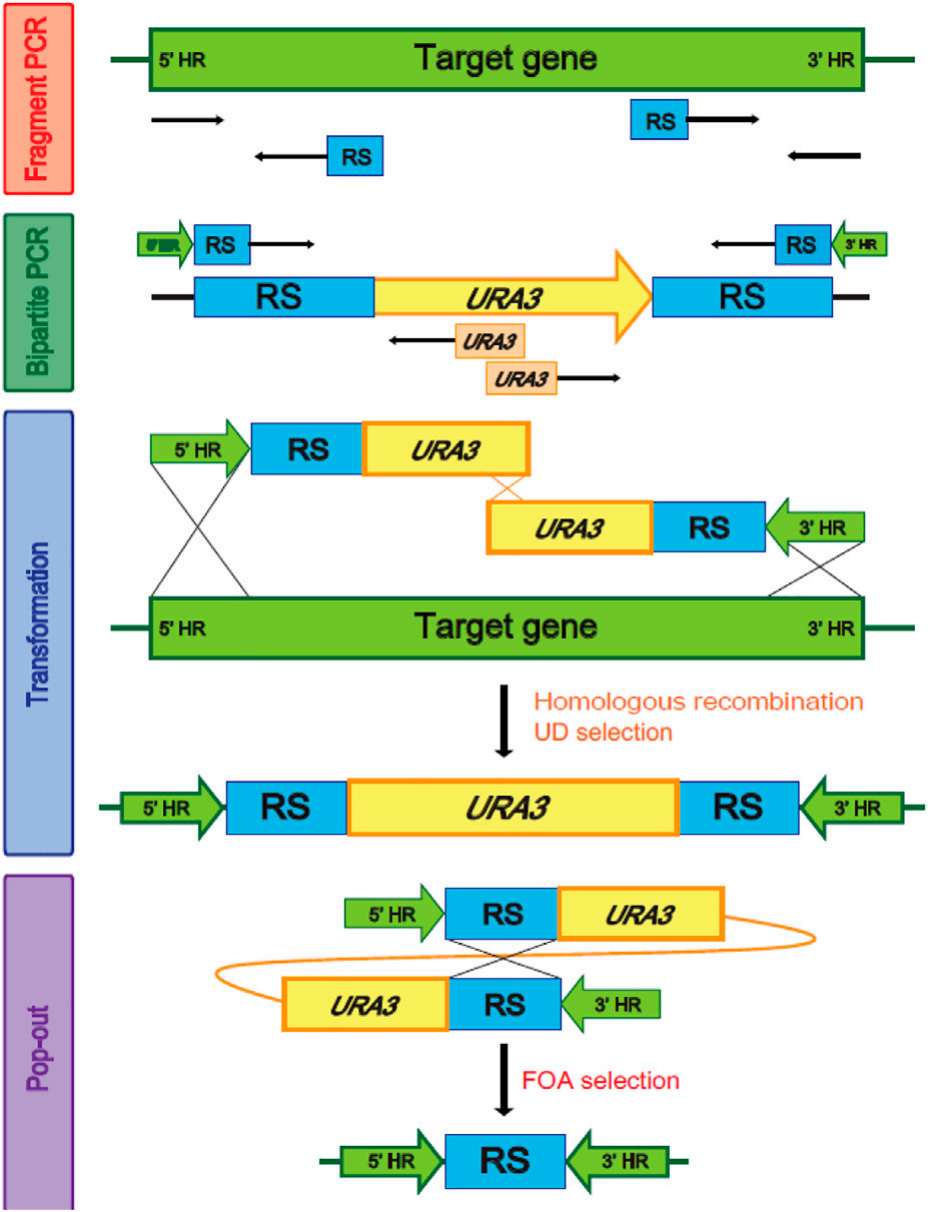
Homologous recombination and the 5-FOA (5-Fluoroorotic acid) selection using *URA3* as a recyclable selection marker. HR, Homologous region, RS, Repeated sequence.

Multiple genes are involved in each step of ω- and β-oxidation (Supplementary Table S2). Therefore, genome-wide modifications are required to engineer a strain of *Y. lipolytica* suitable to produce α,ω-bifunctional monomers with amino residues, aliphatic polymeric monomers with amino residues, and hydrophobic substrates. In *Y. lipolytica,* non-homologous end-joining (NHEJ) is used more than homologous recombination (HR) for the repair of double-strand breaks in DNA (Krtezschmar et al., 2013; Verbeke et al., 2013). To improve HR efficiency, we used the strains with the disrupted *KU70* responsible for NHEJ-based double-strand break repair, and then we performed a bipartite PCR-based gene-targeting method using *URA3* as a recyclable selection marker (Figure 2). The bipartite deletion cassettes used to remove target genes (Supplementary Table S1) were specifically inserted into sites in the genome *via URA3* split-marker recombination. The sites of integration were confirmed by PCR, which was followed by pop-out of *URA3 via* selection of the 5′-FOA plate. This strategy allowed for the deletion of several genes and the construction of various strains possessing ω-/β-oxidation pathways (Table 1)*.*

### 3.2 *Y. lipolytica* platform strains for α,ω-bifunctional monomers with amino residue

To biosynthesize α,ω-bifunctional monomers with amino residues in *Y. lipolytica*, ω-oxidation was combined with ω-transamination reactions. Thus, we intended to oxidize the terminal end of the hydrophobic substrate into an aldehyde group by ω-oxidation and convert the aldehyde group into an amino group by ω-transaminase (ω-TA), which catalyzes the amination between the ω-positioned ketone and amine groups. However, as shown in Figure 1A, ω-oxidation pathway in *Y. lipolytica* oxidizes hydrocarbons substrates such as alkanes, fatty alcohols and acids to their corresponding terminal carboxy function (Craft et al., 2003). Fatty acid synthesized by the ω-oxidation pathway or introduced as a substrate can be further oxidized through the same ω-oxidation pathway to the corresponding α,ω-diacids. Finally, both fatty acids and α,ω-diacids are metabolized through the β-oxidation pathway to yield energy, CO_2_, and water (Poirier et al., 2006).

**FIGURE 3:**
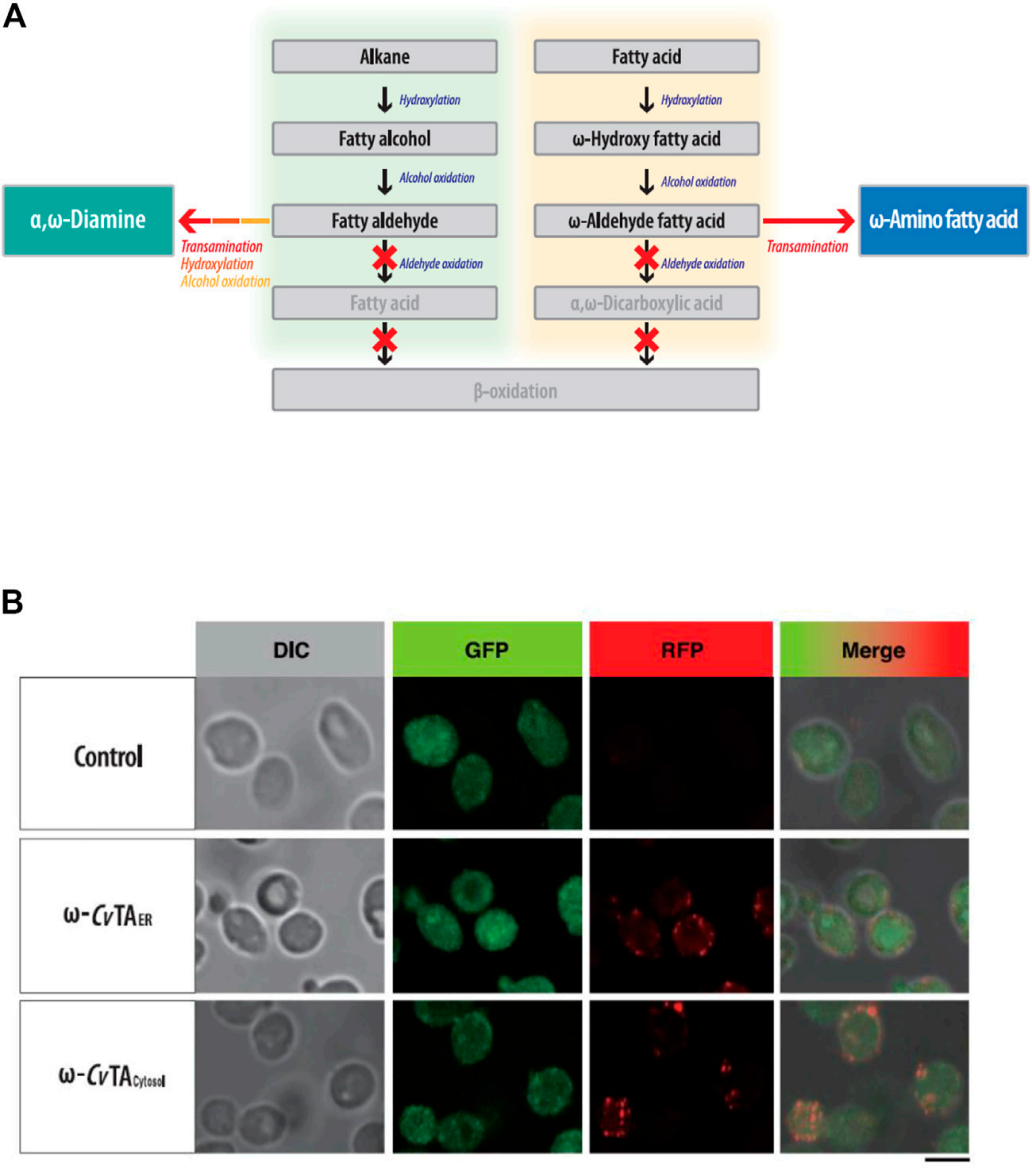
**(A)** Metabolic engineering of *Y. lipolytica* for the biotransformation of hydrocarbons (fatty alkane, alcohol, and acids) into the fatty polymeric monomers with amino residue. **(B)** Subcellular localization of ω-*Cv*TA_ER_ and ω-*Cv*TA_cytosol_ introduced into *Y. lipolytica*. Bar is 5 μm-size.

To block β-oxidation, we consecutively deleted six genes (*POX1*-*6*) encoding the acyl-CoA oxidase. The Δ*POX* strain did not grow on fatty alkanes, fatty alcohols or acids as sole carbon sources (Supplementary Figure S3). To block the oxidizing aldehyde group into the carboxylic group in ω-oxidation, we eliminated four fatty aldehyde dehydrogenase genes (FALDH1-4) and inserted ω-*Cv*TA (Klatte and Wendisch 2015) from *Chromobacterium violaceum* DSM30191 into the genome of the Δ*POX* Δ*FALDH* strain (Figure 3A). The ω-*Cv*TA was placed under the control of *Y. lipolytica-*derived EXP1 promoters (Blazeck et al., 2011), and its expression in two forms was designed with or without the ER targeting leader sequence (ERTLS) (Iida et al., 1998) from *ALK1* at its N-terminus. Confocal microscopy confirmed that two forms of ω-*Cv*TA were successfully expressed in *Y. lipolytica*; and one form (ω-*Cv*TA with ERTLS, ω-*Cv*TA_ER_) was exclusively localized to the ER and the other (ω-TA without ERTLS, ω-*Cv*TA_cytosol_) to the cytosol (Figure 3B).

### 3.3 The effect of multiple gene knock-out on cell growth

**FIGURE 4:**
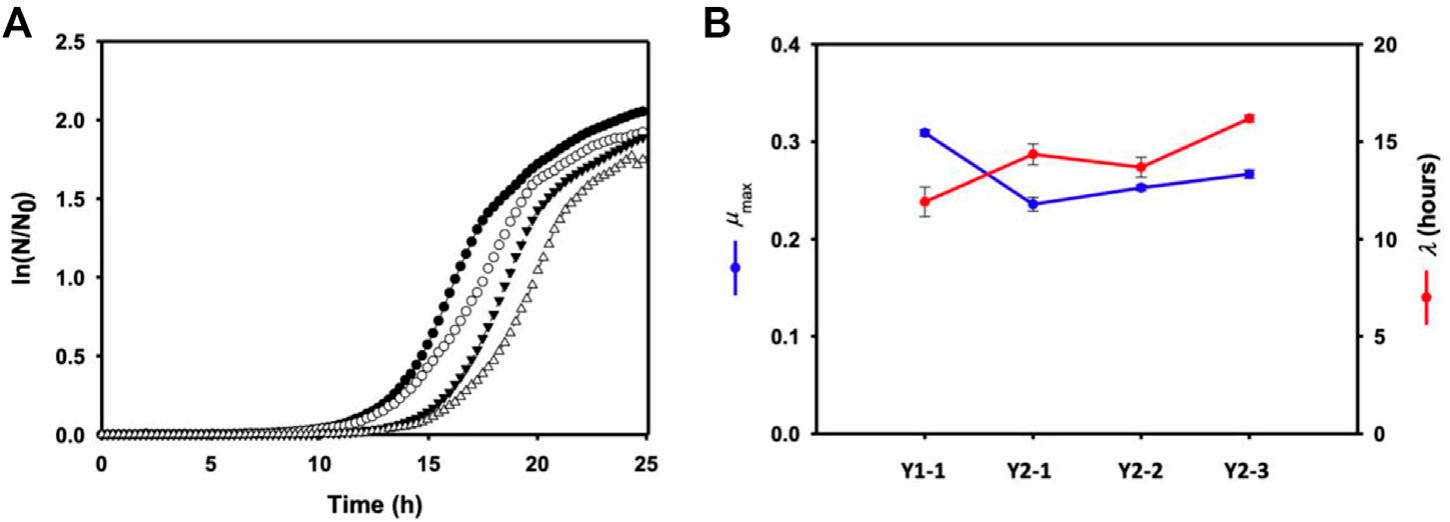
**(A)** Cell growth of each mutant (closed circular: Y1-1, open circular: Y2-1, closed inverted triangle: Y1-1, and open inverted trianlge: Y2-3) in a yeast extract-peptone medium containing 10 g/L yeast extract and 20 g/L Bacto-peptone in sterile 24-well PS plates. **(B)** the maximum specific growth rate (μmax) and lag time (λ) of each mutant calculated by the Gompertz model. Error bars represent the standard error of the mean of at least three independent replicates.

The effect of multiple gene knock-out on cell growth was investigated by monitoring the growth of each mutant using a plate reader. Time-profiles of the monitored cell growth (Figure 4) were fitted to the Gompertz model (Zwietering et al., 1990) by least squares regression, which could determine the maximum specific growth rate (*μ*_max_) and lag time (*λ*) of each mutant. Compared to Pol1g strain, all mutant showed the lower *μ*_max_, representing a theoretical maximum growth rate based on the inflection point of the curve, and more increased *λ*, the adaptation time. However, a remarkable toxic effect on all mutants was not observed.

### 3.4 Production of α,ω-bifunctional monomers with amino residue in *Y. lipolytica*

**FIGURE 5:**
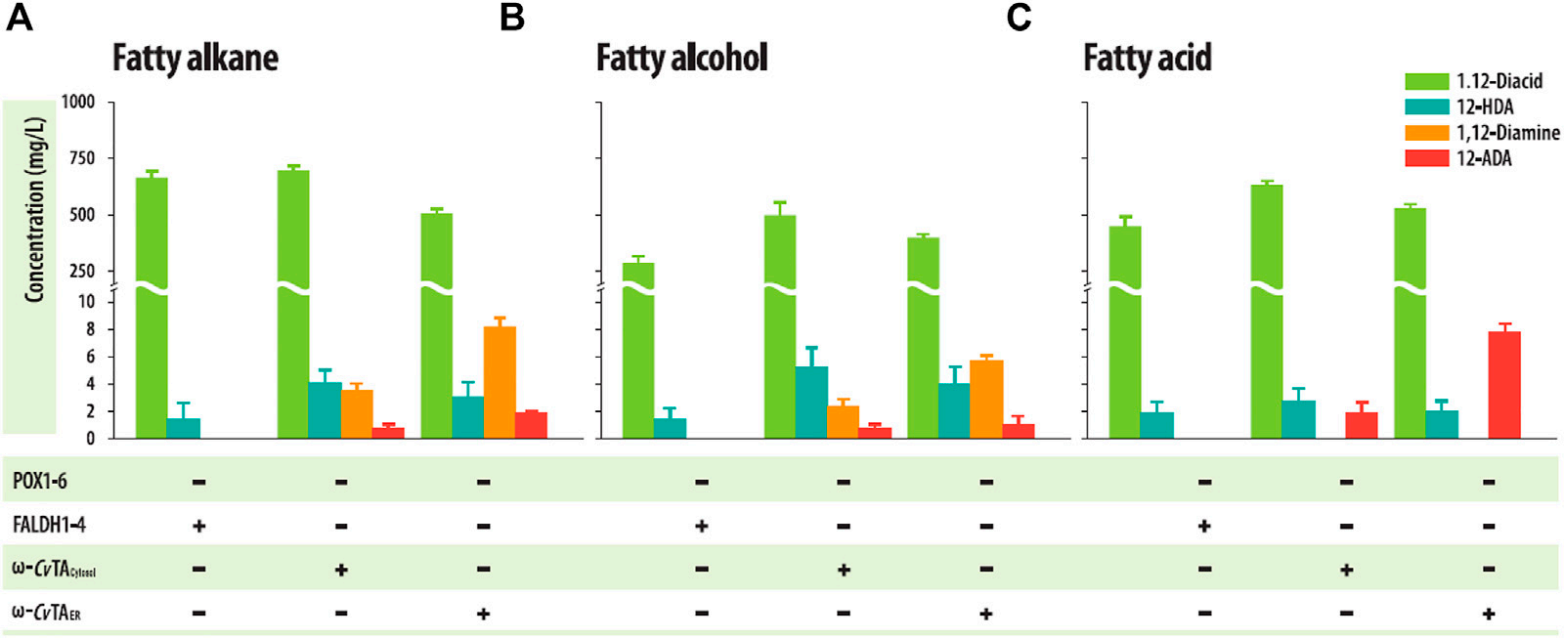
Production of α,ω-diaminododecane and ω-amino dodecanoic acid from **(A)** dodecane, **(B)** dodecanol, and **(C)** dodecanoic acid by engineered *Y. lipolytica* strains in shake flask cultures. Error bars represent the standard error of the mean of at least three independent replicates.

The Δ*POX*Δ*FALDH* strains with ω-*Cv*TA_ER_ or ω-*Cv*TA_cytosol_ successfully accumulated the corresponding 1,12-diaminododecanes into the culture medium in a shaking flask culture with 1 g/L of dodecane or dodecanol as C_12_ hydrocarbon substrates (Figures 5A, B, Supplementary Figure S4), but the strain with ω-*Cv*TA_ER_ produced more than 2-fold of 1,12-diaminododecanes (8.32 ± .70 mg/L, from dodecane as a substrate and 5.81 ± .40 mg/L from dodecanol) than those with ω-*Cv*TA_cytosol_ (3.62 ± .46 mg/L and 2.43 ± .49 mg/L from dodecane and dodecanol, respectively). To examine whether the strains could convert FAs to the corresponding ω-AFAs, these strains were cultivated in a shaking flask with 1 g/L of dodecanoic acid. These strains successfully accumulated 12-aminododecanoic acids in the culture medium (Figure 5C, Supplementary Figure S5). Similar to 1,12-diaminododecanes, the strain with ω-*Cv*TA_ER_ also produced more than 2-folds of 12-aminododecanoic acids (7.94 ± .54 mg/L) than those with ω-*Cv*TA_cytosol_ (1.91 ± .65 mg/L). Surprisingly, 12-aminododecanoic acids were produced from dodecane (1.91 ± .18 mg/L for ω-*Cv*TA_ER_ and .77 ± .28 mg/L for ω-*Cv*TA_cytosol_) and dodecanol (1.11 ± .58 mg/L for ω-*Cv*TA_ER_ and .48 ± .16 mg/L for ω-*Cv*TA_cytosol_) (Figures 4A, B), which may be resulted from the overoxidation of some cytochrome P450s as described previously (Lu et al., 2010). These strains also produced 1,12-dodecanedioic acids and 12-hydroxydodecanoic acids as by-products, and their amounts were similar to those in the Δ*POX* strains.

**FIGURE 6:**
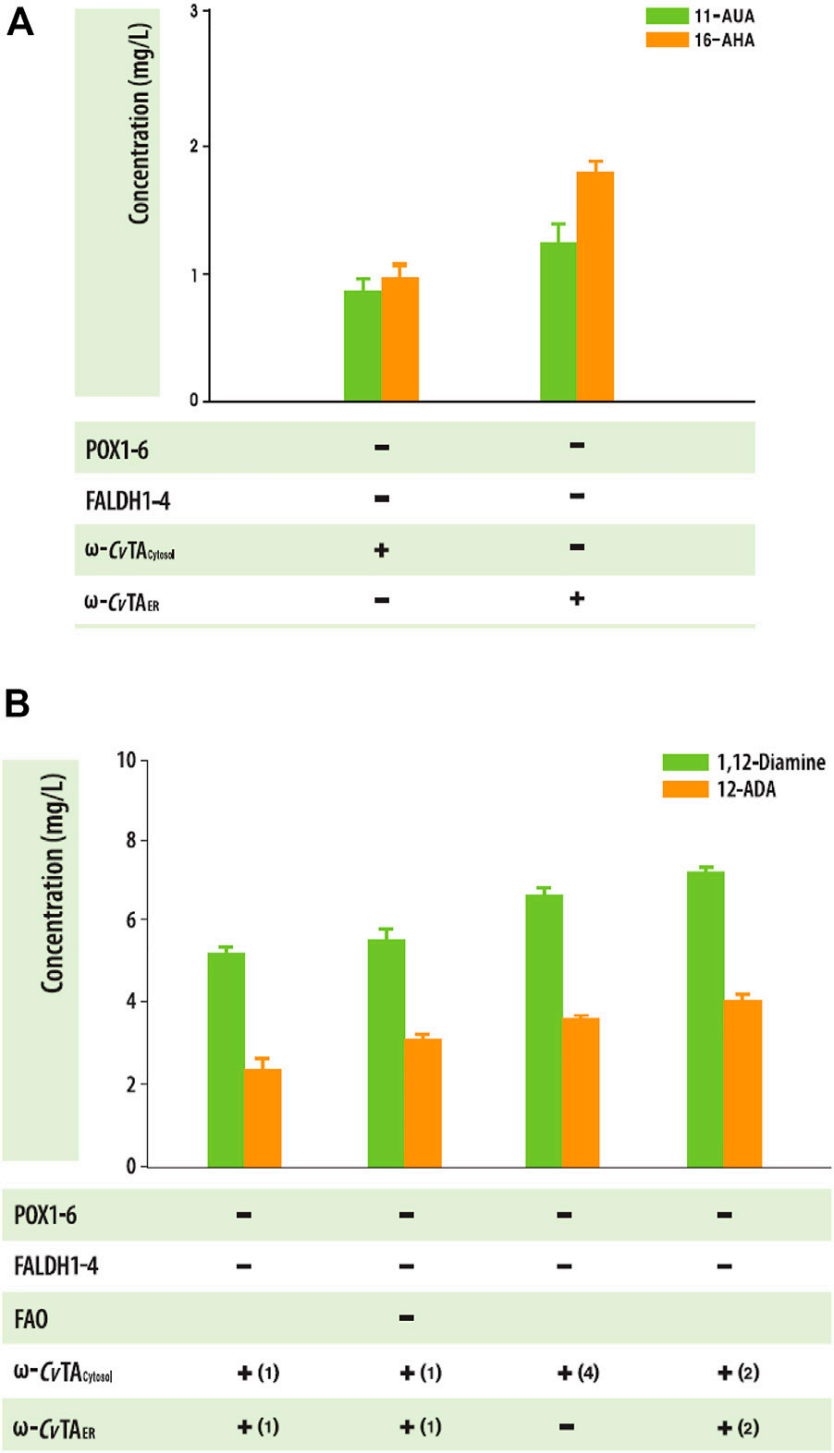
**(A)** Biotransformation of C_11_ and C_16_ fatty acids into the corresponding ω-amino fatty acids in the engineered *Y. lipolytica* strains. **(B)** Effect of Δ*FAO1* and copy numbers of ω-TA on the production of amine-compound production in engineered *Y. lipolytica*. Error bars represent the standard error of the mean of at least three independent replicates.

When substrates were expanded to an odd number (C_11_) or long chain (C_16_), both Δ*POX* and Δ*FALDH* strains with ω-*Cv*TA_ER_ or ω-*Cv*TA_cytosol_ also produced the corresponding ω-AFAs (1.91 ± .18 mg_C11 ω-AFAs_/L and .77 ± .28 mg_C16 ω-AFAs_/L for ω-*Cv*TA_ER_ and 1.91 ± .18 mg_C11 ω-AFAs_/L and .77 ± .28 mg_C16 ω-AFAs_/L for ω-*Cv*TA_cytosol_) (Figure 6A). To increase the production of αω-FDs and ω-AFAs with amino residues, we engineered *Y. lipolytica* strain by knocking out fatty alcohol oxidase and increasing the copy numbers of ω-*Cv*TA. Interestingly, only *FAO1*-deleted strains produced 1,12-diols, indicating that *FAO1* plays an important role in the degradation of alcohol groups, which increased the production of α,ω-diamines and ω-amino fatty acids (Figure 6B). These findings were similar with previous studies (Gatter et al., 2014; Iwama et al., 2015).

## 4 Discussion

Biotransformation of hydrocarbons into polymeric monomers with amino residues has mainly been attempted using whole-cell systems of *E. coli* by introducing heterologous CYP genes. However, attempts encountered some problems, such as CYP expression and hydrophobic substrate uptake in *E. coli*-based whole-cell biotransformation. In particular, the correct expression of CYP in *E. coli* requires a heme precursor such as expensive δ-ala-leuvenic acid (Wu et al., 2006; Ichinose and Wariishi 2013). The *n-*alkane-assimilating yeasts with an endogenous CYP belonging to the CYP52 family and a reductase provide an advantageous approach for ω-oxidation of hydrocarbons. The diploid yeast *C. tropicalis* produces 174 g/L C_14_ diacid from methyl ester C_14_ fatty acid and 153 g/L of C_13_ α,ω-diacids from tridecane (Picataggio et al., 1992). Cathay Biotechnologies Inc. (China) used *C. tropicalis* to industrially produce C_11_–C_16_ α and ω-diacids from petroleum-based *n*-paraffin. Lu et al. (2010) reported high-level production of ω-hydroxy fatty acids using *C. tropicalis* through a modification of the α- and β-oxidation pathways.

In this study, we needed genome-wide modification and thus selected an alternative *n-*alkane-assimilating haploid *Y. lipolytica* as a starting strain since *C. tropicalis* is an obligate diploid (Seevai et al., 2013). We successfully generated α,ω-diamines and ω-amino fatty acids from hydrocarbons by oxidizing the terminal of the hydrophobic substrate into an aldehyde group *via* ω-oxidation and converting an aldehyde group into an amino group by ω-transaminase, which catalyzes the amination between the ω-positioned ketone and amine group. To the best of our knowledge, this study is the first to establish a *Y. lipolytica*-based biocatalytic system for the production of α,ω-diamines or ω-amino fatty acids from *n*-alkanes, fatty alcohols and acids. Although whole-cell catalytic systems using *E. coli* has been recently developed (Yoo et al., 2022), the systems have the critical disadvantages including CYP expression and hydrophobic substrate uptakes. Firstly, CYP expression in *E. coli* requires a heme precursor, which supplements culture medium with δ-ala-leuvenic acid, an expensive commodity (Wu et al., 2006; Cheesman et al., 2013; Ichinose and Wariishi, 2013; Ichinose et al., 2015). Furthermore, *E. coli* shows limited uptake of hydrophobic substrates, which can lower the productivity.

**FIGURE 7:**
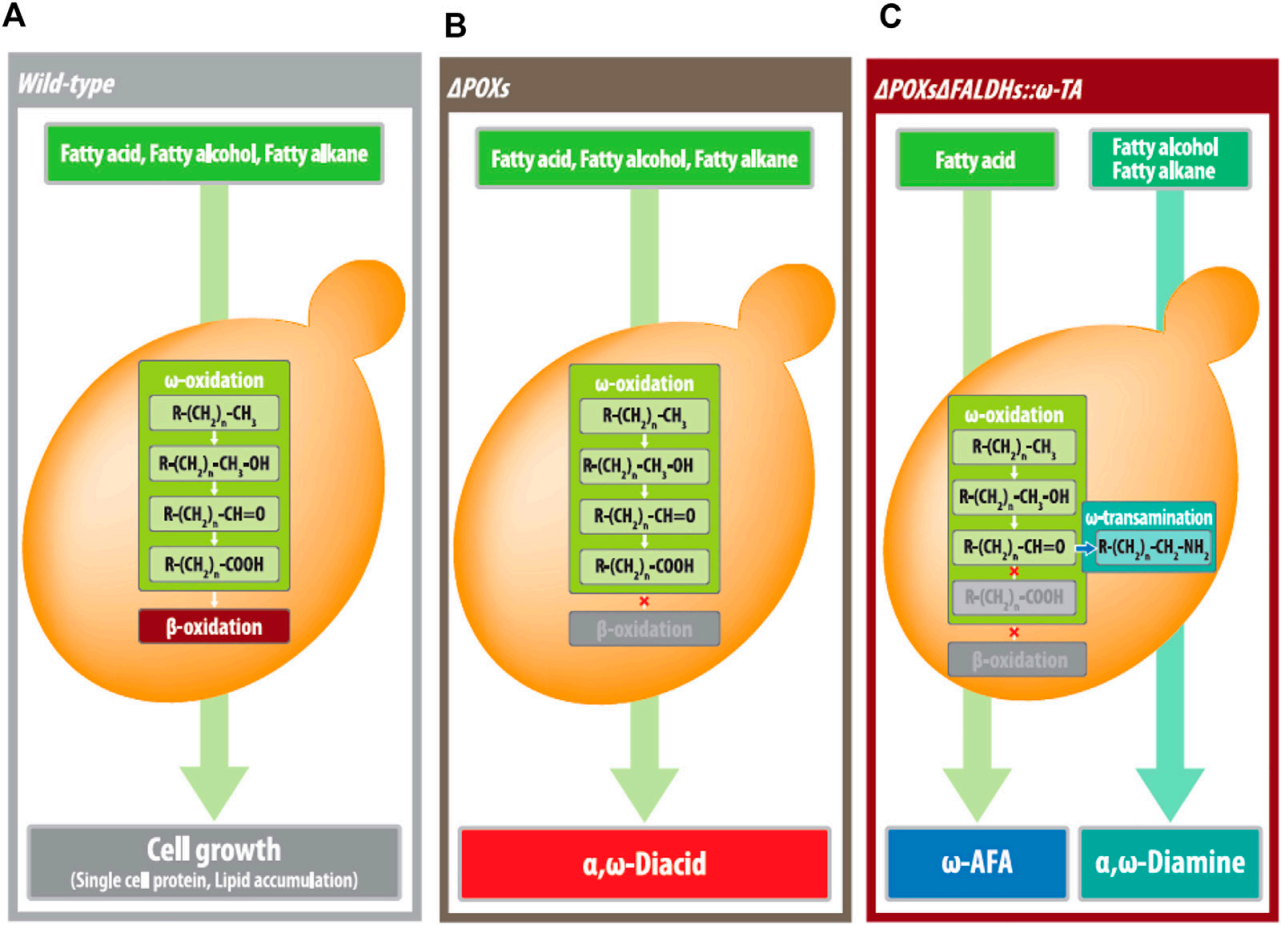
Yeast biocatalytic platforms for biotransfomation of MCLC hydrophobic substrates to industrial applicable products including **(A)** single cell protein or lipid by wild-type strain, **(B)** α,ω-diacids (fatty alkanes, fatty alcohols, and fatty acids as substrates), by the Δ*POXs* strains*,* **(C)** ω-amino fatty acids (for fatty acids as substrates), and α,ω-diamines (for fatty alkane and fatty alcohol as substrates) by the Δ*POXs* Δ*FALDHs* strains expressing ω-TA.

In conclusion, with the commercially available α,ω-diacid bioprocess, this yeast biosynthesis that produces medium- and long-chain α,ω-diamines and ω-amino fatty acids could complete the yeast platform technology generating all medium- and long-chain aliphatic polyamide monomers, α,ω-biofunctionalized with one or both of carboxylic acid or amino residues (Figure 7). Therefore, although the use of plant oils has been focused on manufacturing biodiesel, this study will contribute in advancing the inexpensive plant oil-derived fatty acids or alcohols as substrates while producing high-value products [for example, from dodencanoic acid or dodecanol (US$ 1–1.5/kg) to 1,12-dodecanedioc acid, 1,12-dodecanediamine, and 1,12-dodecanediol (above US$ 7/kg)]. In this study, however, the low production of α,ω-diamines and ω-amino fatty acids was limited due to over-oxidized by-products (1,12-diacids and 12-hydroxy fatty acids). In our future studies, we will conduct the engineering of the CYP protein to reduce over-oxidation activity and develop fermentation and purification processes for α,ω-diamines and ω-amino fatty acids.

## Data Availability

The original contributions presented in the study are included in the article/Supplementary Material, further inquiries can be directed to the corresponding authors.
