# Supplementary material for: Biosynthesis of aliphatic plastic monomers with amino residues in Yarrowia lipolytica
Source: Front Bioeng Biotechnol. 2023 Jan 11;10:825576. doi: 10.3389/fbioe.2022.825576 (PMC9875067; doi:10.3389/fbioe.2022.825576)
Supplement: Supplementary file 1 [file DataSheet1.docx]

Supplementary Material

## Supplementary Figures


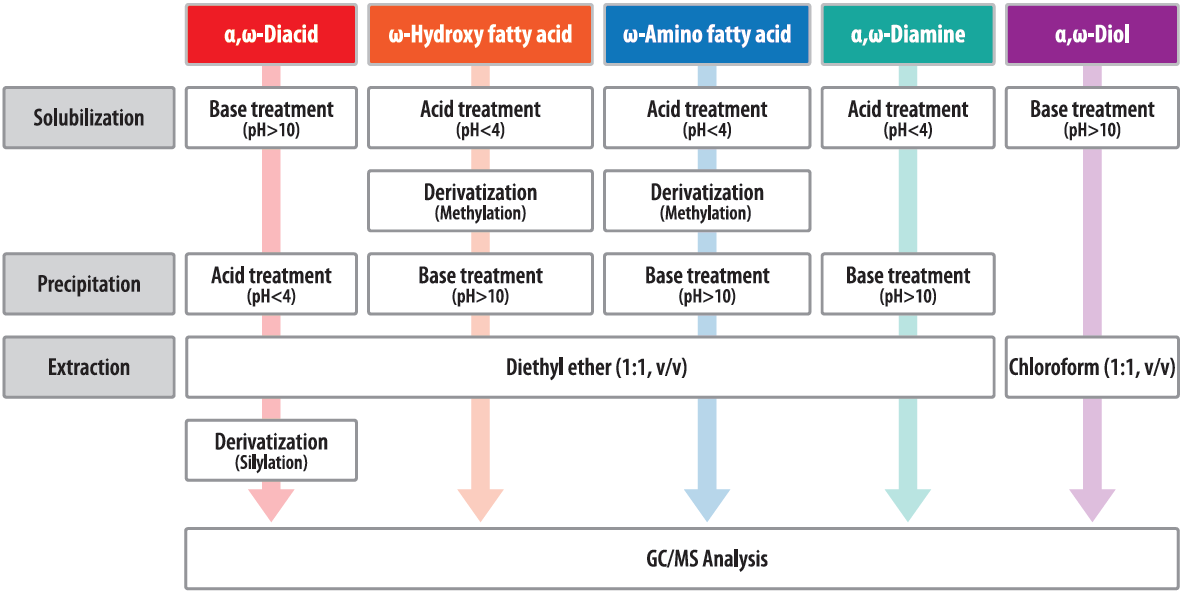


**Figure S1.** Sample preparation of α,ω-bifunctional chemicals for GC-MS analysis.


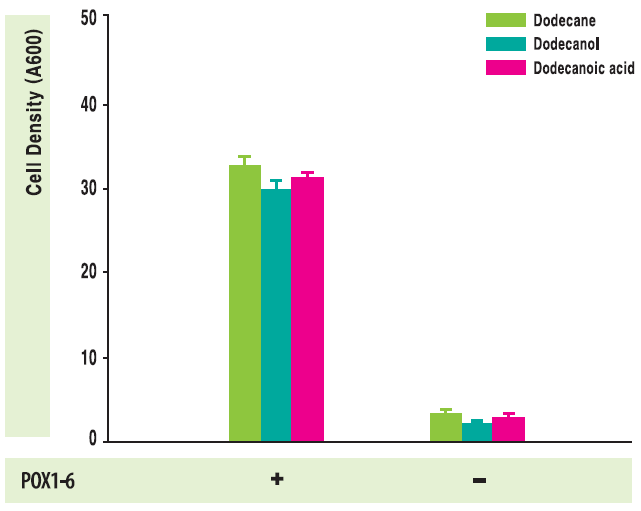


**Figure S2.** Cell growth of wild-type and β-oxidation-deleted *Y. lipolytica* strains on fatty alkanes, fatty alcohols, and fatty acids as sole carbon sources. *Y. lipolytica* strains were pre-grown in a test tube with 3 mL YPD (glucose 20 g/L, yeast extract 10 g/L, Bacto peptone 20 g/L) media overnight at 30 °C with shaking at 200 rpm. Then, the cultures were inoculated into 500 mL baffled-flask with 50 mL of growth medium (pH 6.0) containing 20 g/L dodecane or dodecanol or dodecanoic acid, 5 g/L yeast extract, 6.7 g/L YNB w/o amino acids, 5 g/L (NH_4_)_2_SO_4_, and 0.05 g/L uracil. The cultures were incubated for 48 h at 30 °C with the shaking at 200 rpm, and the cell densities were measured after incubation.


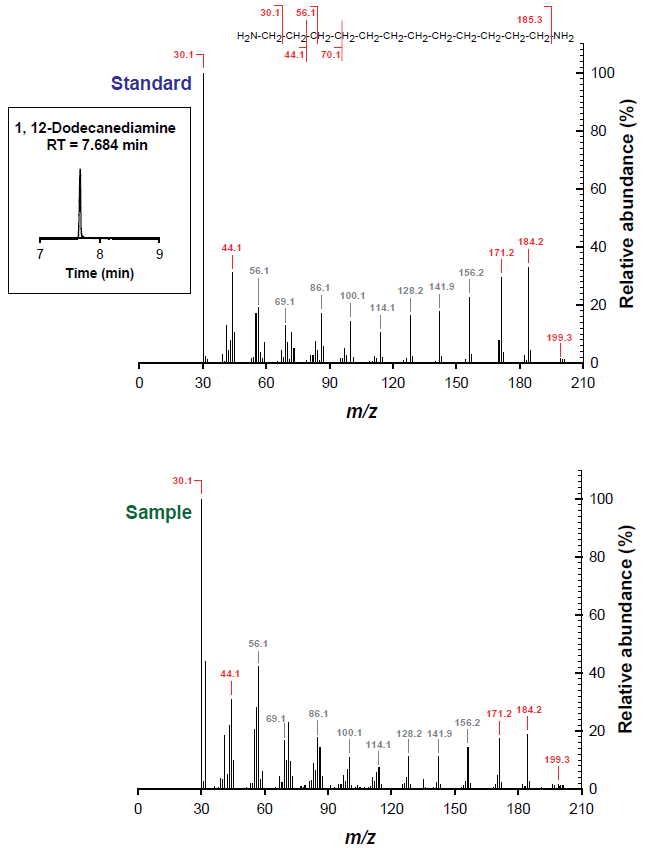


**Figure S3.** GC-MS analysis of 1,12-dodecanediamine from standard and culture broth.


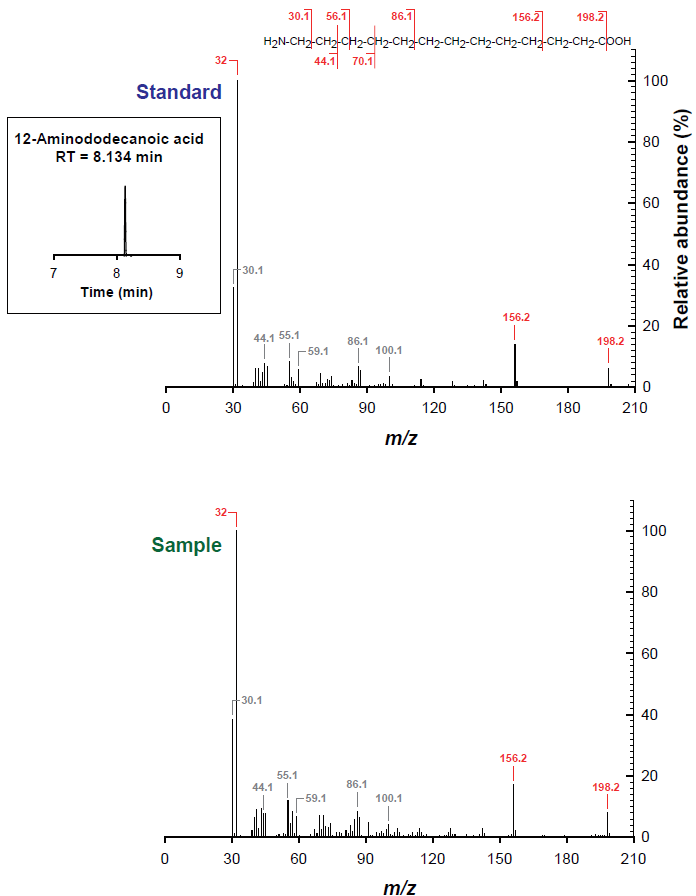


**Figure S4.** GC-MS analysis of 12-aminododecanoic acid from standard and culture broth.

## Supplementary Table

Table S1. Primers used in this study. Sequences that are complementary to the genomic DNA of *Y. lipolytica* are written in underlined.

| **Primers** | **Sequences (5’⭢3’)** | **Description** |
| --- | --- | --- |
| HisG1-BglII F | aattgggcccagatctcagaccggttcagacaggat | pop-out vector cloning |
| HisG1-EcoRI R | tctctgggcggaattcggaggtgcggatatgaggta |  |
| HisG1-NotI F | tgtttctcggcggccgccagaccggttcagacaggat |  |
| HisG1-BamHI R | tccaacgcgtggatccggaggtgcggatatgaggta |  |
| HisG2-BglII F | aattgggcccagatctaacgctacctcgaccagaaa |  |
| HisG2-EcoRI R | tctctgggcggaattctcttctcgatcggcagtacc |  |
| HisG2-NotI F | tgtttctcggcggccgcaacgctacctcgaccagaaa |  |
| HisG2-BamHI R | tccaacgcgtggatcctcttctcgatcggcagtacc |  |
| glt1-BglII F | aattgggcccagatcttcagaacttgcgccgataaa |  |
| glt1-EcoRI R | tctctgggcggaattcctttgccagctagaccatagag |  |
| glt1-NotI F | tgtttctcggcggccgctcagaacttgcgccgataaa |  |
| glt1-BamHI R | tccaacgcgtggatccctttgccagctagaccatagag |  |
| glt2-BglII F | aattgggcccagatctattggcgggttcgttactt |  |
| glt2-EcoRI R | tctctgggcggaattccctggaagaaggccgtattatc |  |
| glt2-NotI F | tgtttctcggcggccgcattggcgggttcgttactt |  |
| glt2-BamHI R | tccaacgcgtggatcccctggaagaaggccgtattatc |  |
| POX1-F1 | ttcctcaatggtggagaaga | *ACO1* deletion |
| POX1-R1 | tctttatcctgtctgaaccggtctggtaccatagtccttgccatgc |  |
| POX1-F2 | atcgctacctcatatccgcacctcccttctgtcccccgagtttct |  |
| POX1-R2 | aagaagggcttgagagtcg |  |
| POX2-F1 | cccaacaacactggcac | *ACO2* deletion |
| POX2-R1 | tctttatcctgtctgaaccggtctgctcctcatcgtagatggc |  |
| POX2-F2 | atcgctacctcatatccgcacctccgacaagacccgacaggc |  |
| POX2-R2 | agaccagagtcctcttcg |  |
| POX3-F1 | accttcacagagccaccca | *ACO3* deletion |
| POX3-R1 | atggctctctgggcggtgttgggggtgttgatgatg |  |
| POX3-F2 | ttgttgtgtttctcgcaaggttctcatcgaggcctg |  |
| POX3-R2 | aggaaaggtcgaagagtgctct |  |
| POX4-F1 | actgcgagagcgatctg | *ACO4* deletion |
| POX4-R1 | tctttatcctgtctgaaccggtctgttcatgagcatgtagtttcg |  |
| POX4-F2 | atcgctacctcatatccgcacctccgaggacgacaaagccggag |  |
| POX4-R2 | agagcagagtcctcctcaa |  |
| POX5-F1 | aacttcctcacaggcagcgagc | *ACO5* deletion |
| POX5-R1 | atggctctctgggcggagtagagagtgggagttgaggtc |  |
| POX5-F2 | ttgttgtgtttctcgccccgtcaaggacgctgag |  |
| POX5-R2 | acagtaaggtggggcttgactc |  |
| POX6-F1 | agtccctcaacacgtttaccg | *ACO6* deletion |
| POX6-R1 | tctttatcctgtctgaaccggtctgccatttagtggcagcaacgtt |  |
| POX6-F2 | atcgctacctcatatccgcacctccgagctctgatcaaccgaacc |  |
| POX6-R2 | aggaagggtctaatgacaga |  |

Table S1. Continued

| **Primers** | **Sequences (5’⭢3’)** | **Description** |
| --- | --- | --- |
| FALDH1-F1 | aatcactcctcctacgc | *FALDH1* deletion |
| FALDH1-R1 | tctttatcctgtctgaaccggtctgtggtctcggggacacctc |  |
| FALDH1-F2 | atcgctacctcatatccgcacctccccatcatcaagccccgaa |  |
| FALDH1-R2 | accgacataatctgagcaat |  |
| FALDH2-F1 | accactaggtgagatcgag | *FALDH2* deletion |
| FALDH2-R1 | tctttatcctgtctgaaccggtctgctccgacactaccggaacgc |  |
| FALDH2-F2 | atcgctacctcatatccgcacctcccttgctcccacagttgtt |  |
| FALDH2-R2 | gatcacccagaaccatagc |  |
| FALDH3-F1 | gtgacccccaccacgtcac | *FALDH3* deletion |
| FALDH3-R1 | tctttatcctgtctgaaccggtctgttctgacattttcagcgccac |  |
| FALDH3-F2 | atcgctacctcatatccgcacctccccattacgagcgtttgacgg |  |
| FALDH3-R2 | cagggctggggaccacc |  |
| FALDH4-F1 | taccgactggaccagattc | *FALDH4* deletion |
| FALDH4-R1 | tctttatcctgtctgaaccggtctgcggcagtggcaatgatcttac |  |
| FALDH4-F2 | atcgctacctcatatccgcacctccgactcgattcatcgctcctac |  |
| FALDH4-R2 | caaatctttcggaagattcgg |  |
| HISG1-F | cagaccggttcagacaggat | pop-out cassette |
| HISG1-R | ggaggtgcggatatgaggta |  |
| HISG2-F | aacgctacctcgaccagaaa |  |
| HISG2-R | tcttctcgatcggcagtacc |  |
| glt2-F | tcagaacttgcgccgataaa |  |
| glt2-R | ctttgccagctagaccatagag |  |
| glt3-F | attggcgggttcgttactt |  |
| glt3R | cctggaagaaggccgtattatc |  |
| EXP1-F | ccaagcttggtaccgagctcagagtttggcgcccgttttttc | ω-TA vector cloning |
| EXP1-R | cgttgtttttgcatatgtgctgtagatatgtcttgtgtgtaa |  |
| TEF-F | ccaagcttggtaccgagctcaaactttggcaaagaggctgca |  |
| TEF-R | cgttgtttttgcatatgtttgaatgattcttatactcagaag |  |
| ALK1-F | ccaagcttggtaccgagctcagatctgtgcgcctctacagaccc |  |
| ALK1-R | cgttgtttttgcatatgagtgcaggagtattctggggagga |  |
| XPR2t-F2 | gtcgacgcaattaacagatagtttgccg |  |
| XPR2t-R3 | ctcgagggatcccggaaaacaaaacacgacag |  |
| TA-F | catatgcaaaaacaacgtactacctccc |  |
| TA-R | gtcgacttaggccaaaccacgggctttc |  |
| ATATG2-ER-F | actcctgcactcatatgtccaacgccctcaacctg |  |
| XTATG2-ER-F | ccaatccaacacatatgtccaacgccctcaacctg |  |
| ER-R-1 | cgttgtttttgcatagaaccgccaccgccgctaccgccaccgcccgaaccgccaccgccgaatcgtgaaatatccttgggct |  |
| ER-R-2 | cgttgtttttgcatatgagaaccgccaccgccgctaccgccaccgcccgaaccgccaccgccgaatcgtgaaatatccttgggct |  |
| ETATG2-ER-1 | tgattacgccaagcttgagtttggcgcccgttttttc |  |
| ETATG2-ER-2 | acaggttgagggcgttggacatatgtgctgtagatatgtcttgtgtgtaa |  |
| TTATG2-ER-1 | tgattacgccaagcttaaactttggcaaagaggctg |  |
| TTATG2-ER-2 | acaggttgagggcgttggacatatgtttgaatgattcttatactcagaag |  |
| ER-F | atgtccaacgccctcaacctg |  |
| ER-R-3 | cgttgtttttgcatagaaccgccaccgccgctac |  |
| TA-FALDH4-F1 | taccgactggaccagattc | ω-TA cassette |
| TA-FALDH4-R1 | cggcagtggcaatgatcttac |  |
| TA-FALDH4-F2 | ctcctctatggtctagctggcaaagactcgattcatcgctcctac |  |
| TA-FALDH4-R2 | caaatctttcggaagattcgg |  |
| ATATG2-F | gtcggtaagatcattgccactgccgagatctgtgcgcctctacagac |  |
| ETATG2-F | gtcggtaagatcattgccactgccggagtttggcgcccgttttttc |  |
| TTATG2-F | gtgggtaagatcattgccactgccgaaactttggcaaagaggctgc |  |
| XTATG2-F | gtcggtaagatcattgccactgccgacgcgtggagagtttgggtt |  |
